# Development of a neural network predicting signals for time-domain diffuse optical tomography

**DOI:** 10.1007/s13534-026-00578-9

**Published:** 2026-04-18

**Authors:** Shu Horie, Hidenobu Yajima, Makito Abe, Masayuki Umemura

**Affiliations:** 1 Center for Computational Sciences, University of Tsukuba, Ten-nodai, 1-1-1, Tsukuba, Ibaraki 305-8577 Japan; 2 Faculty of Natural Sciences, National Institute of Technology, Kure College, Agaminami, 2-2-11, Kure, Hiroshima 737-8506 Japan

**Keywords:** Diffuse optical tomography, Radiative transfer, Numerical simulations, Machine learning

## Abstract

Time-domain diffuse optical tomography (TD-DOT) is a powerful method for diagnosing anomalies in biological tissue such as brain hemorrhage and tumor. However, numerical simulations for TD-DOT require exploring a large number of parameter combinations and demand substantial computational resources. To address this challenge, we develop a neural network (NN) that can rapidly infer time-resolved signals from given tissue parameters. A high-quality training dataset for the NN is generated using ray-tracing-based radiative transfer simulations for 640 different absorber parameter combinations. Using the simulation data, we utilize NN to construct an emulator reproducing time-resolved signals for any parameters not used in the training data. We train two NN models with different training datasets: one with Gaussian noise added and the other without Gaussian noise. The NN trained with noisy data demonstrates superior performance, accurately reproducing time-resolved signals for unseen parameters. Its errors remain comparable to the noise level in the training data, highlighting strong robustness and generalization capability. Each inference takes only $\sim 2\times 10^{-3}$ seconds, which is $>10^6$ times faster than a direct radiative transfer simulation. This drastic speedup suggests the potential for efficient inverse problem analysis and application in real-time clinical diagnosis.

## Introduction

Diffuse Optical Tomography (DOT) is a useful technique to acquire information in biological tissue [1–4]. In DOT, near-infrared (NIR) light in the range of $\sim 700 - 1000 \, \textrm{nm}$ is utilized. Although the majority of photons are scattered and absorbed within biological tissue by hemoglobin and water, a small fraction can emerge from the body. This optical property of NIR light enables it to penetrate biological tissue to a depth of several centimeters. By illuminating biological tissue with NIR light and analyzing the transmitted or reflected light emerging from the surface, it is possible to non-invasively obtain spatial information about tissue composition. Since a relatively large amount of NIR photons are absorbed in anomalies such as bleeding sites and malignant tumors than in normal regions, the status of anomalies (e.g. position, size, etc.) influences the light emerging from the biological surface. This capability allows for the detection of anomalies without the risk for radiative exposure. Both experimental and computational studies have been conducted to advance the clinical applications of DOT, particularly for the diagnosis of conditions such as brain hemorrhage and malignant tumors [5–10].

In DOT, continuous-wave (CW) measurements utilize only intensity information to infer the spatial distribution of absorbers, typically under the assumption of a known scatterer distribution within the tissue. Frequency-domain (FD) DOT, on the other hand, makes use of both intensity and phase information, allowing for the reconstruction of both absorption and scattering coefficients by analyzing the photons at different modulation frequencies [11, 12]. Similarly, time-domain (TD) DOT provides information on the spatial distribution of absorption and scattering coefficients, as the temporal profiles of detected signals are influenced by these optical properties [11, 13]. Recently, an experimental setup for TD-DOT with picosecond-order temporal resolution has been developed [14]. This advancement facilitates the reconstruction of high-resolution optical property maps of biological tissue using TD-DOT.

To reconstruct the optical properties of biological tissue from TD-DOT signals, it is essential to solve photon propagation under multiple scattering within the tissue, which is known as the forward problem. To this end, the radiative transfer equation (RTE) must be solved, as it describes the time evolution of photon intensity. However, the RTE with scattering and absorption is an integro-differential equation with no general analytical solution, necessitating numerical approaches for its resolution. Direct numerical solutions of the RTE with scattering and absorption are computationally expensive in terms of both processing time and memory. This high cost arises from the iterative procedures required for convergence, as well as the multi-dimensional nature of the intensity phase space, which includes position, direction, and time. To mitigate these challenges, alternative approaches such as the Monte Carlo method and diffusion approximation have been employed [15–19]. The Monte Carlo method, while widely used, introduces statistical noise due to its stochastic nature, whereas the diffusion approximation is valid only in the regime of frequent photon scattering, where radiation fields are nearly isotropic. Recently, Yajima et al. [20] developed TRINITY (Time-dependent Radiative transfer In Near-Infrared TomographY), a numerical simulation code that directly solves the time-dependent RTE including both scattering and absorption. The time-resolved signals generated from TRINITY show good agreement with those measured in phantom experiments. Furthermore, Takamizu et al. [21] demonstrated that a neural network trained on TRINITY simulation results can successfully predict the position of an absorber using phantom experiment data. These findings indicate that TRINITY can provide high-quality signals without the need for phantom experiments. TRINITY employs the Message Passing Interface (MPI) and Open Multi-Processing (OpenMP) for parallel computation and utilizes a ray-tracing method to accurately model scattering and absorption along predefined rays. Since this approach minimizes numerical diffusion, it enables precise estimation of optical time-resolved signals in biological tissue. Furthermore, Abe et al. (submitted) improved the TRINITY code by incorporating wavelet transformations, which adequately and automatically reduces the number of rays and thus accelerates the computation of scattering interactions. As a result, TD-DOT simulations with TRINITY provide high-quality data. However, despite these optimizations, high-resolution simulations with TRINITY are still time consuming, making them unsuitable for real-time clinical diagnosis.

A promising approach for achieving prompt diagnosis in DOT is the application of machine learning to solve the inverse problem, i.e., the reconstruction of the optical properties of biological tissue. This reconstruction is particularly challenging due to the non-linear relationship between optical properties and light measurements, which arises from multiple scattering effects. Machine learning techniques have been widely explored for reconstructing optical properties in DOT. For instance, Takamizu et al. [21] trained a deep learning model based on Long Short-Term Memory (LSTM) networks to detect the position of cancer from simulated TD-DOT data. Similarly, Dale et al. [22] employed FDU-Net (a hybrid architecture combining Fully-connected, encoder-Decoder, and U-Net modules [23]) to reconstruct absorption and scattering coefficients from simulated FD-DOT data. Beyond direct reconstruction, another potential application of machine learning in DOT is the development of a predictive model that estimates light measurements for arbitrary configurations of absorbers within biological tissue. Such a model could address the practical limitation that real-time numerical simulations are computationally infeasible for clinical diagnosis. If an accurate machine learning-based forward model can be constructed, it could be integrated with the Markov Chain Monte Carlo (MCMC) method to infer the probability density function of tissue features that best explain observed light measurements.

Brain hemorrhage and tumor are critical diseases that necessitates early detection for timely intervention, as it remains a major cause of mortality worldwide [24–28]. Accurate diagnosis of bleeding and cancer requires determining their several key characteristics including its location, size, shape, and absorption coefficient. Consequently, radiative transfer simulations designed for anomaly diagnosis must account for a vast parameter space encompassing numerous possible feature combinations. Developing a machine learning model that emulates the outcomes of numerical radiative transfer simulations represents a promising approach to facilitating early detection of anomalies. However, constructing such a model remains a significant challenge due to the complexity of light-tissue interactions and the high-dimensional nature of the parameter space.

In this study, we develop a machine learning-based emulator for time-dependent radiative transfer simulations for DOT. Our emulator is constructed using a neural network with fully connected layers. To evaluate its performance, we conduct numerical simulations of anomalies like brain hemorrhage and tumor near the brain surface as a representative model. For simplicity, we focus on three key features of absorbers (i.e., the anomaly): horizontal position, depth, and size. The neural network is formulated as a regression model, taking these features as input and predicting the corresponding time-resolved light signals detected at measurement points.

## Methods

### Radiative transfer simulations

#### Simulation setup

**Fig. 1:**
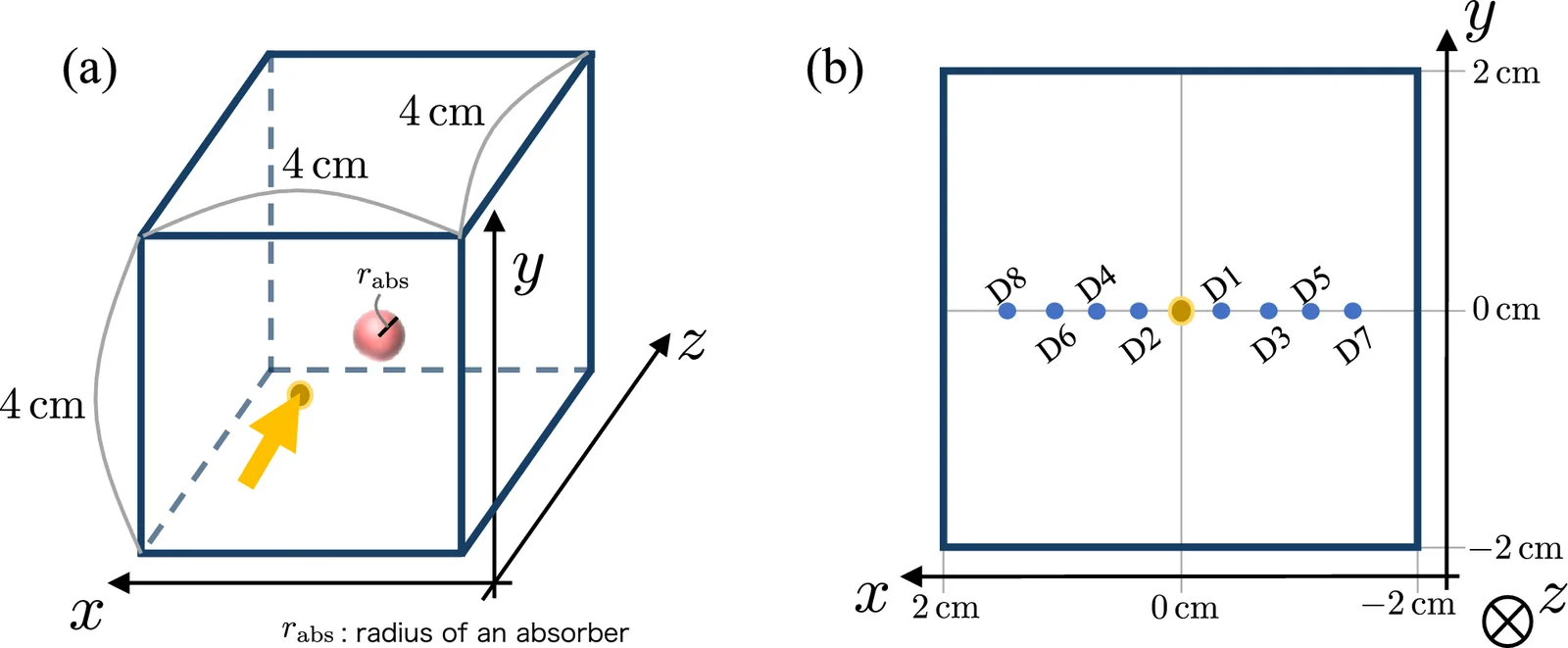
**a** Schematic image of the simulation box and coordinates. The pulse is perpendicularly injected from the center of the *x-y* plane (yellow point and arrow). The pulse (yellow point and arrow) is perpendicularly injected from the center of the *x-y* plane ($z=0.0\,\textrm{cm}$ plane, i.e. the origin of the coordinates). A single spherical absorber with a radius $r_\textrm{abs}$ is included in the simulation box. **b** Positions of the eight detectors. Each detector (blue point) is located along $y=0.0\,\textrm{cm}$ on the pulse injected surface. The detectors are $0.3\,\textrm{cm}$ apart from each other and the pulse injected point

In this study, we simulate pulse propagation in a cubic domain designed to model anomalies like brain hemorrhage and tumor near the brain surface and analyze the light emerging from the surface after undergoing multiple scattering and absorption. The simulation domain consists of a $4.0\,\textrm{cm} \times 4.0\,\textrm{cm} \times 4.0\,\textrm{cm}$ cube and is discretized into $128^3$ cells (Fig. 1). A single absorber, representing the anomaly, is placed within the domain and is assumed to have a spherical shape. For the simulations, we employ the radiative transfer code TRINITY [20], which accurately solves the RTE without relying on the diffusion approximation. Furthermore, recent enhancements by Abe et al. [29] have incorporated wavelet transformations into TRINITY, significantly improving computational efficiency. Using the updated TRINITY code, we compute the time evolution of the radiation field by numerically solving the following RTE

1 $$\begin{aligned}&\frac{1}{v(\boldsymbol{r})}\frac{\partial I(t,\boldsymbol{r},\boldsymbol{\Omega })}{\partial t} + \boldsymbol{\Omega }\cdot \boldsymbol{\nabla }I(t,\boldsymbol{r},\boldsymbol{\Omega }) \nonumber \\&= -\{\mu _a(\boldsymbol{r})+\mu _s(\boldsymbol{r})\}I(t,\boldsymbol{r},\boldsymbol{\Omega }) \\&\quad+ \mu _s(\boldsymbol{r}) \oint _{\Omega '} I(t,\boldsymbol{r},\boldsymbol{\Omega }')p(\boldsymbol{\Omega }',\boldsymbol{\Omega }) \textrm{d}\boldsymbol{\Omega }' \end{aligned}$$

where *t* is time, $\boldsymbol{r}$ is position, $\boldsymbol{\Omega }$ is direction (solid angle), $v(\boldsymbol{r})$ is the speed of light in the tissue, $I(t,\boldsymbol{r},\boldsymbol{\Omega })$ is the specific intensity, $\mu _a(\boldsymbol{r})$ and $\mu _s(\boldsymbol{r})$ are the absorption and scattering coefficients, respectively, and $p(\boldsymbol{\Omega }',\boldsymbol{\Omega })$ is a phase function. We assume a uniform refractive index of *n=1.511* throughout the domain, independent of the absorber properties, yielding a speed of light in tissue given by *v=c/n* where *c* is the speed of light in vacuum. The absorption coefficient $\mu _a$ differs between the background tissue and the absorber. For background (i.e. no anomaly area), we set $\mu _a=0.3\,\mathrm {cm^{-1}}$, while the absorber has ten times higher coefficient than the background ($\mu _a=3.0\,\mathrm {cm^{-1}}$). The scattering coefficient is fixed at $\mu _s=80.0\,\mathrm {cm^{-1}}$ for both the background and the absorber [30, 31]. The phase function $p(\boldsymbol{\Omega }',\boldsymbol{\Omega })$ describes the angular redistribution of scattered light, characterizing the transition from the incoming direction $\boldsymbol{\Omega }'$ to outgoing direction $\boldsymbol{\Omega }$ at position $\boldsymbol{r}$. For this study, we adopt the Henyey-Greenstein phase function:

2 $$\begin{aligned} p(\boldsymbol{\Omega },\boldsymbol{\Omega }') = \frac{1}{4\pi }\frac{1-g^2}{(1+g^2-2g\boldsymbol{\Omega }'\cdot \boldsymbol{\Omega })^{3/2}} \end{aligned}$$

with an anisotropy factor of *g=0.9*. The time evolution is computed from *t=0* to 3 nanoseconds ($\textrm{ns}$).

**Fig. 2:**
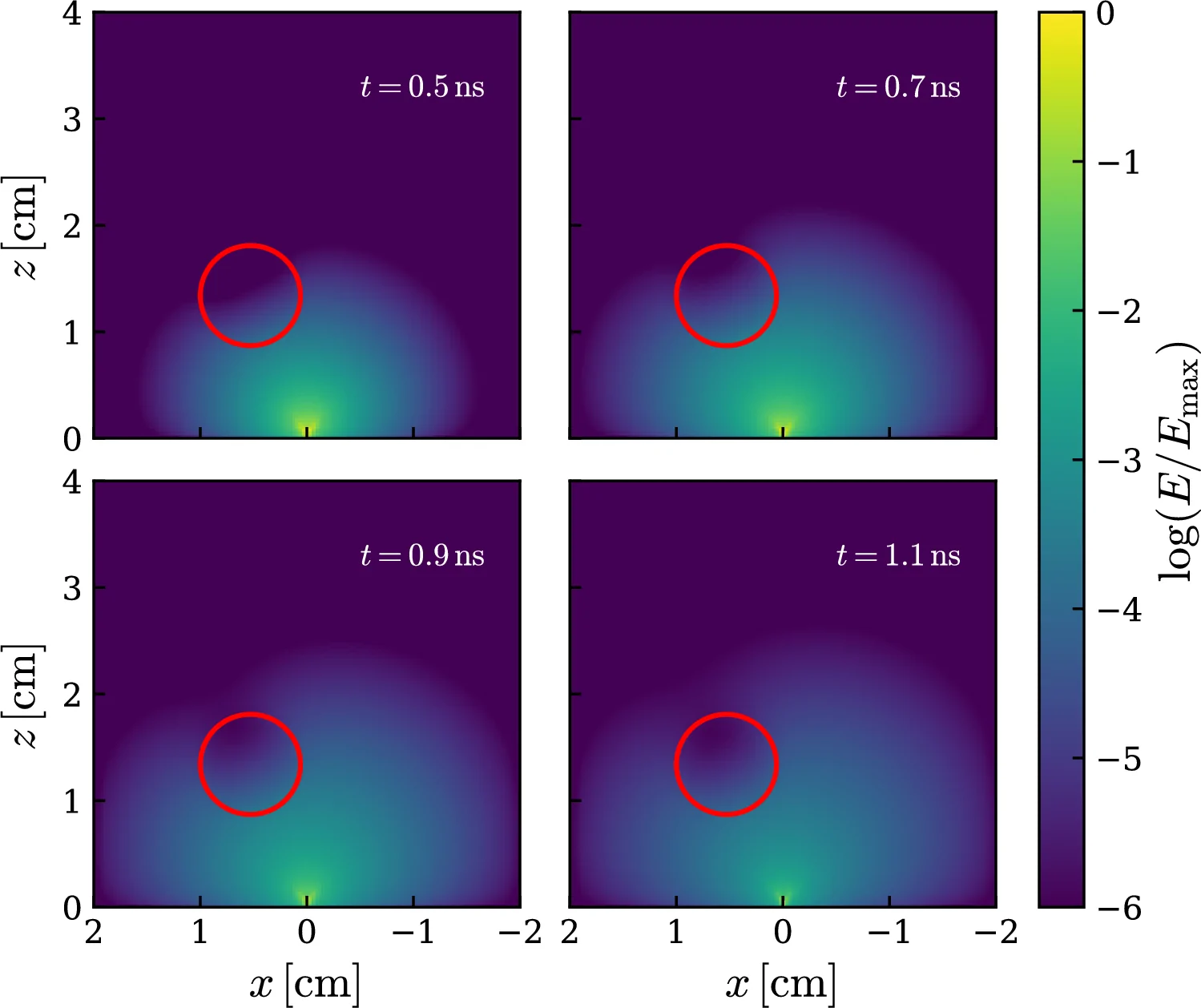
Example snapshots of the photon energy density at $t=0.5, \ 0.7, \ 0.9, \ 1.1$ nanoseconds ($\textrm{ns}$) on the $y=2.0\,\textrm{cm}$ plane. The absorber is located at $(x, z)=(0.53, 1.34)\,\textrm{cm}$ with a radius of $0.47\,\textrm{cm}$ and is indicated by the red circles. Due to the presence of the absorber, the energy density is reduced in its vicinity. The energy density is normalized by the maximum value at $t=0.9\,\textrm{ns}$

For the calculation of specific intensity in the time domain, we input the pulse used in Yajima et al. [20] from the center of a surface of the simulation box and eight detectors are set (see Fig. 1). Photons propagate through the simulation domain with undergoing scattering and absorption and are subsequently detected on the same surface where the pulse is injected. Figure 2 shows an example of the time evolution of the photon energy density on the $y=0.0\,\textrm{cm}$ plane. Light injected at $y=0.0\,\textrm{cm}$ propagates through the simulation box, undergoing multiple scattering and absorption events. The presence of a absorber reduces the local energy density around it. We arrange eight detectors (D1-8) along a horizontal line, each spaced $0.3\,\textrm{cm}$ apart from both the pulse injection point and adjacent detectors, as illustrated in Fig. 1 (b). In detecting light signals, we adopt the same detector opening angle of $\sim 15^\circ$ as in previous studies and obtain the signals in the same manner [20, 29]. Photons that go outside the simulation domain are not reintroduced into the system.

#### Specific intensity and absorption measure

For training our neural network, we first calculate the normalized intensity $I_\textrm{norm}^i(t)$ at the *i*-th detector, defined as

3 $$\begin{aligned} I_\textrm{norm}^i(t) = \frac{I_\textrm{abs}^i(t)}{I_\textrm{noabs}^{i,\textrm{max}}}, \end{aligned}$$

where $I_\textrm{abs}^i(t)$ represents the intensity of the outgoing light toward the *i*-th detector in the case with an absorber as a function of time, while $I_\textrm{noabs}^{i,\textrm{max}}$ is the maximum intensity at the same detector when no absorber is present. Each intensity is estimated by integrating specific intensity over solid angles. Additionally, we calculate the absorption measure $A^i(t)$ at the *i*-th detector, defined as

4 $$\begin{aligned} A^i(t) = \frac{I_\textrm{abs}^i(t)}{I_\textrm{noabs}^i(t)} - 1, \end{aligned}$$

where $I_\textrm{noabs}^i(t)$ is the intensity detected at the *i*-th detector in the case without any absorber as a function of time. Since the absorption measure $A^i(t)$ exhibits a more pronounced variation across the different absorber features compared to the normalized intensity $I_\textrm{norm}^i(t)$, $A^i(t)$ is considered to be more suitable for diagnostic purposes using our neural network [21]. We use both $I_\textrm{norm}^i(t)$ and $A^i(t)$ to train our neural network model.

#### Features in simulations

Due to the optical properties of the absorber and the simulation setup described above, the radiation field depends on the position and size of an absorber. Consequently, the light emerging from the surface of the simulation box is also a function of the position and size of the absorber. In this work, we run radiative transfer simulations using 640 different sets of the three features of the absorber for simplicity: horizontal direction $x_\textrm{abs}$, depth $z_\textrm{abs}$, and radius $r_\textrm{abs}$, where $x_\textrm{abs}$ and $z_\textrm{abs}$ represent the center coordinates of the absorbers and we assume that absorbers are spherical with their centers located on the $y=0.0\,\textrm{cm}$ plane. We note that, in this study, we intentionally restrict the absorber properties to these three features, although other factors, such as the vertical position $y_\textrm{abs}$, the absorption coefficient $\mu _\textrm{a}$, the absorber shape, and the presence of multiple absorbers, can also significantly influence the temporal evolution of $I_\textrm{norm}$ and *A*. Developing a neural network model that can rapidly and accurately infer time-resolved signals while accounting for all these factors is a highly challenging task, primarily due to the computational complexity of solving the RTE for a large parameter space. We therefore focus on this reduced set of features to establish a tractable and well-controlled framework, and extensions to include additional absorber properties will be explored in future work.

**Fig. 3:**
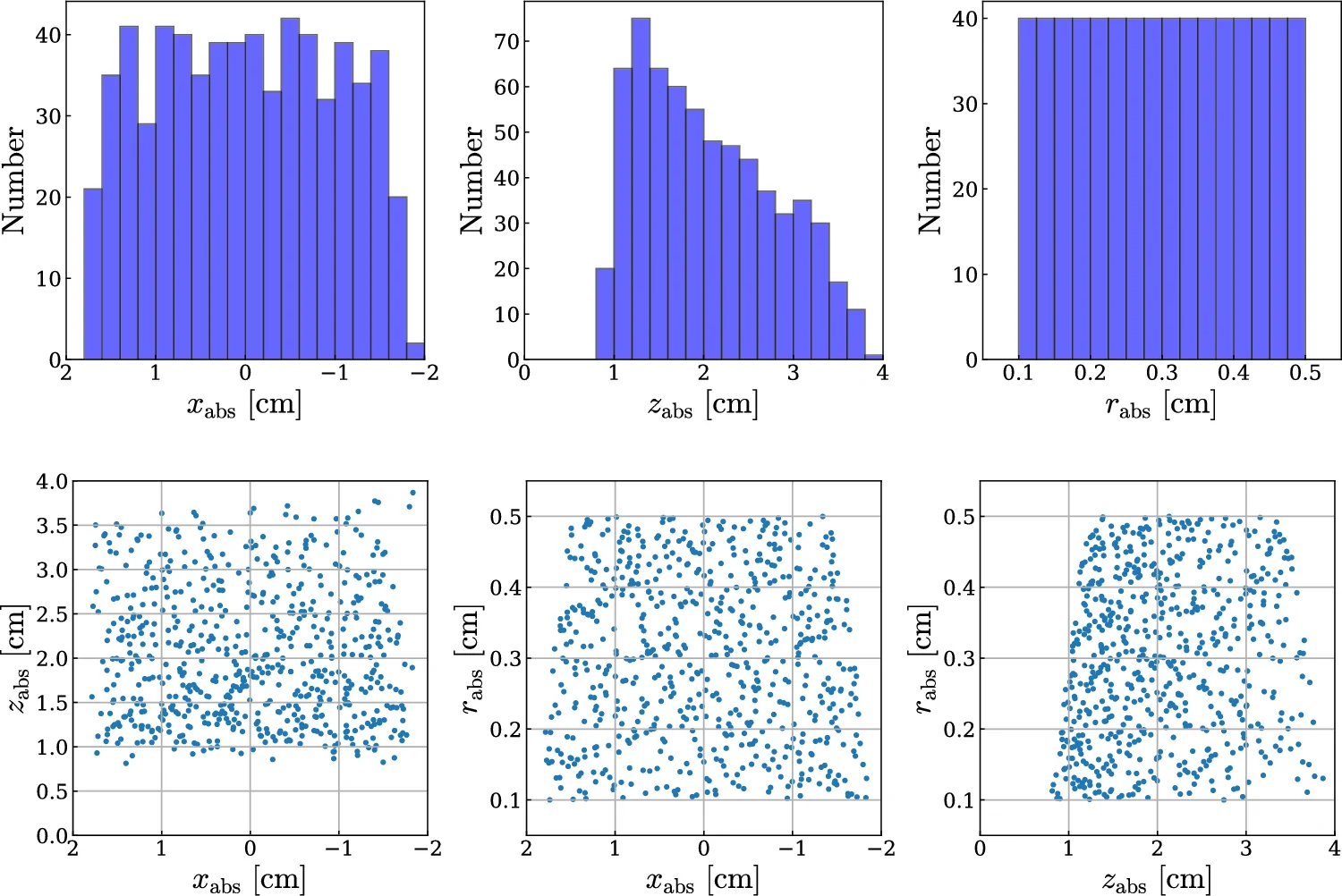
Histograms (top) and scatter plots (bottom) of 640 sets of the features (horizontal direction $x_\textrm{abs}$, depth $z_\textrm{abs}$, and radius $r_\textrm{abs}$) used in our simulations. Each simulation includes a single absorber whose position and radius are determined by this distribution

We randomly generate the sets of the features using the Latin hypercube sampling (LHS), which is a statistical technique that allows for the generation of a quasi-random set of parameter values from a multidimensional probability distribution [32, 33]. LHS is particularly suited for randomly sampling *N* parameter combinations from an *M*-dimensional parameter space with a small bias. The feature sets are generated as follows. First, we generate 640 pseudo-uniform random numbers from a 3D space, in which each variable is *∈ [0,1]*, using LHS. The numbers are then converted into the three features of absorbers following the inverse transform method. The radius $r_\textrm{abs}$ is set so as to follow a continuous uniform distribution between $0.1\,\textrm{cm}$ and $0.5\,\textrm{cm}$. The horizontal direction $x_\textrm{abs}$ follows a continuous uniform distribution between $-2.0\,\textrm{cm}$ and $2.0\,\textrm{cm}$ so that the absorber does not extend beyond the simulation box for a given radius $r_\textrm{abs}$. For each value of $r_\textrm{abs}$, the depth $z_\textrm{abs}$ is sampled from a probability density function following a truncated exponential distribution, with values ranging between 0.7 and $4.0\,\textrm{cm}$ to prevent the absorber from protruding beyond the box. For simplicity, we neglect the optical effect of the skull in our simulations since its optical properties do not differ significantly from those of brain tissue [34], although we consider the absorber to represent an anomaly site located behind the skull. For the exponential distribution, we choose the parameter of $\lambda = 2.0\,\textrm{cm}$, which characterizes the scale length of the distribution and concentrates more absorbers at $z \lesssim 2.0\,\textrm{cm}$. This choice is motivated by our observation that both the normalized intensity $I_\textrm{norm}^i(t)$ and the absorption measure $A^i(t)$ exhibit minimal sensitivity to absorbers located at $z_\textrm{abs} \gtrsim 2.0\,\textrm{cm}$, while they vary more significantly when $z_\textrm{abs} < 2.0\,\textrm{cm}$. Therefore, the neural network should focus on learning this sensitive region. Figure 3 shows the distribution of the features of the absorbers in the parameter space.

Using the 640 sets of features, we perform simulations with a single absorber placed in the simulation box and obtain the optical values of $I_\textrm{norm}^i(t)$ and $A^i(t)$ at each detector.

### Neural network

#### Fully-connected neural network model

**Fig. 4:**
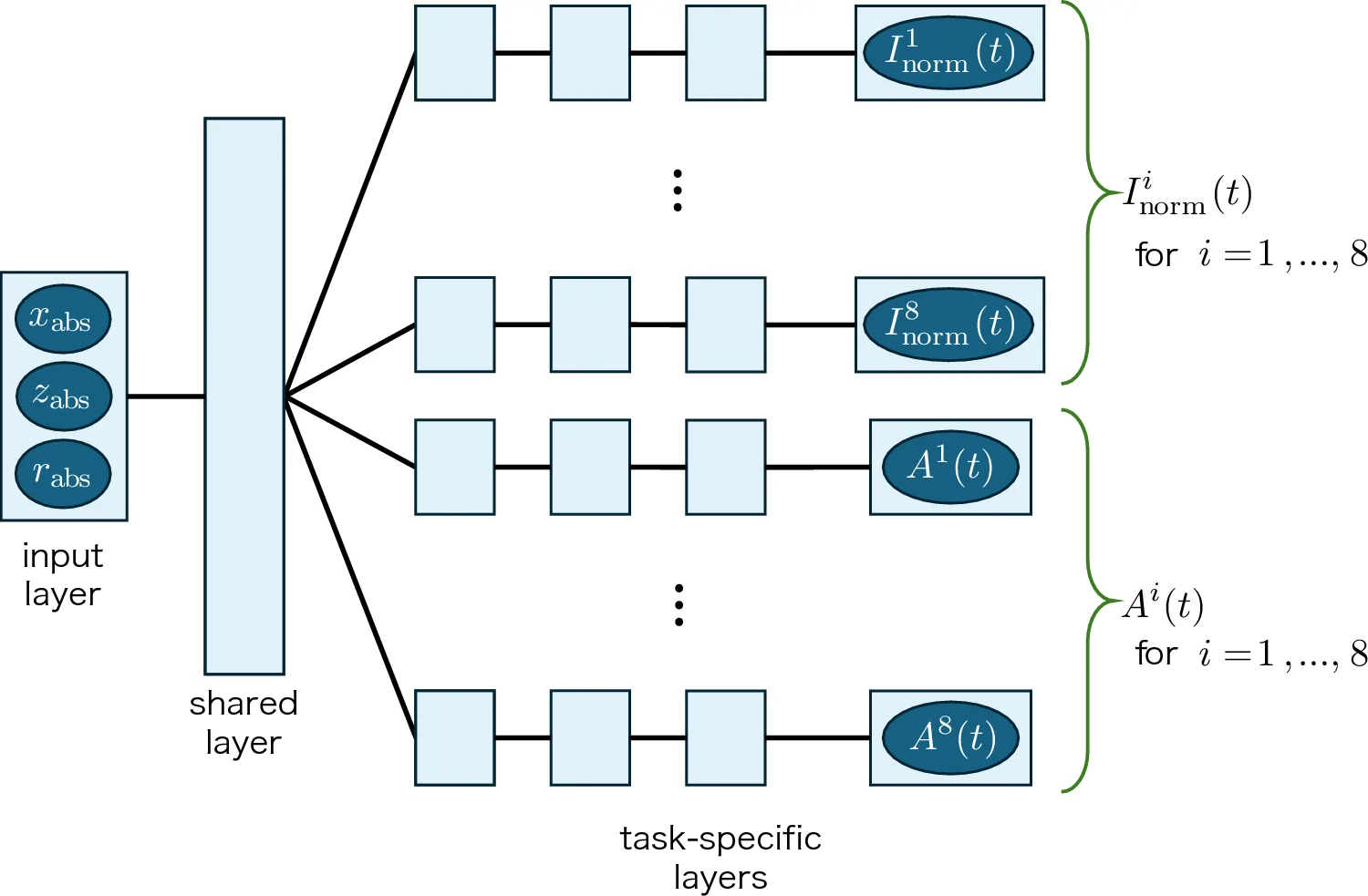
Schematic image of our fully-connected neural network using deep multi-task learning. The input layer receives three absorber features: the horizontal position $x_\textrm{abs}$, depth $z_\textrm{abs}$, and radius $r_\textrm{abs}$. The shared hidden layer and the three hidden layers in the task-specified layers have 32 neurons. The activation function for the shared layer is ReLU. The activation functions for the three hidden layers in the task-specific layers are Tanh. The output layers consist of $N_\textrm{dim}^I = 254$ neurons for predicting the discrete-time evolution of the normalized intensity $I_\textrm{norm}^i(t)$ and $N_\textrm{dim}^A = 476$ neurons for the absorption measure $A^i(t)$ at each detector (*i = 1, … , 8*)

We employ the open-source python library PyTorch [35] to develop a neural network model that predicts the normalized intensity $I_\textrm{norm}^i(t)$ and the absorption measure $A^i(t)$ at each detector from a given set of the features of an absorber. Figure 4 illustrates the schematic image of our fully-connected neural network model, which employs deep multi-task learning. The network architecture consists of a single input layer, a shared hidden layer, and task-specific layers.

The input layer of the network receives the three absorber features: the horizontal position $x_\textrm{abs}$, depth $z_\textrm{abs}$, and radius $r_\textrm{abs}$. The shared hidden layer use the ReLU activation function. The task-specific layers contain three hidden layers, all using Tanh activation, and output layers. Each layer in the shared and task-specified layers has 32 neurons. The output layers consist of $N_\textrm{dim}^I = 254$ neurons for predicting the discrete time evolution of $I_\textrm{norm}^i(t)$ and $N_\textrm{dim}^A = 476$ neurons for $A^i(t)$. The weights in our network are initialized randomly following a Xavier normal distribution [36]. These weights are updated iteratively through forward and backward propagation to minimize the loss function, using the Adam optimizer [37] with a learning rate of 0.001. The training process involves 500 epochs, with a batch size of 8. Although we have run simulations from *t=0* to $3\,\textrm{ns}$, we focus on partial time ranges around the peaks of $I_\textrm{norm}^i(t)$ and $A^i(t)$ since the peaks are strongly influenced by the features. Specifically, we employ the time ranges from *t=0.4* to $1.2\,\textrm{ns}$ for $I_\textrm{norm}^i(t)$ and from *t=1.0* to $2.5\,\textrm{ns}$ for $A^i(t)$. These time ranges correspond to the numbers of output dimensions of $N_\textrm{dim}^I=254$ for $I_\textrm{norm}^i(t)$ and $N_\textrm{dim}^A=476$ for $A^i(t)$, respectively. The loss function is computed as the sum of the mean squared error (MSE) of $I_\textrm{norm}^i(t)$ and $A^i(t)$, defined as

5 $$\begin{aligned} MSE&= \sum _{i=1}^8 \Biggl [ \frac{1}{N_\textrm{dim}^I} \sum _{j=1}^{N_\textrm{dim}^I} \left( I_\textrm{norm}^{i,NN}(t_j) - I_\textrm{norm}^{i,true}(t_j) \right) ^2 \nonumber \\&\qquad \quad + \frac{1}{N_\textrm{dim}^A} \sum _{j=1}^{N_\textrm{dim}^A} \left( A^{i,NN}(t_j) - A^{i,true}(t_j) \right) ^2 \Biggr ] \end{aligned}$$

where the superscripts *NN* and *true* refer the values produced by our neural network and the true values, respectively.

#### Data for training, validation, and test

We train our network using two types of datasets: one without noise and another with added noise. The 640 datasets obtained from the simulations are first used for training and validation. To validate our neural network model, we perform *k*-fold cross-validation to determine the optimal network architecture and hyperparameters described above (e.g. number of neurons, learning rate, etc.). In this cross-validation, we employ *k=5*, meaning that the datasets are randomly divided into 5 subsamples. One subsample is assigned as the validation set, while the remaining four are used for training. This process is repeated 5 times, with each subsample serving as the validation set once. For the noiseless data, we validate network architectures and hyperparameters straightforwardly following this cross-validation process. On the other hand, for the data with noise, we augment each training subsample tenfold through duplication and then add a Gaussian noise with a standard deviation of *σ =0.01* to the signals of $I_\textrm{norm}^i(t)$ and $A^i(t)$. We calculate the loss using Eq. 5 in each process at the 100th epoch for each fold. After testing various architectures and hyperparameter combinations, we find that both datasets yield the lowest average loss with the same network configuration. In addition, we find that incorporating the shared layer significantly improves the network performance, suggesting that this layer plays an important role in the prediction process. In the main results, we use the optimized neural network model to predict time-resolved signals. After finalizing the network architecture and the hyperparameters, we retrain the networks using all 640 datasets. The training processes take approximately 16 min for the network trained with noiseless data and 2.3 h for the network trained using data with noise on a single Intel Xeon Gold 6140 CPU core. We compare the performance of the network based on these different data handling methods.

For test data, we generate an additional 100 datasets by running radiative transfer simulations using feature sets that follow the same probability distribution as that used for the training data. The inference time of the trained machine learning model is approximately $2\times 10^{-3} \, \textrm{sec}$ when executed on a single Intel CPU. In the results section, we show the performance of our neural network model by comparing the signals predicted by our network with the true signals from the test data.

## Results

### Predicting time-resolved signals

Our neural network predicts the time-resolved signals of the normalized intensity $I_\textrm{norm}^i(t)$ and the absorption measure $A^i(t)$ at each detector. To evaluate the performance of our model, we compare the predicted signals with the true signals of the test data.

#### Neural network trained using data without noise

**Fig. 5:**
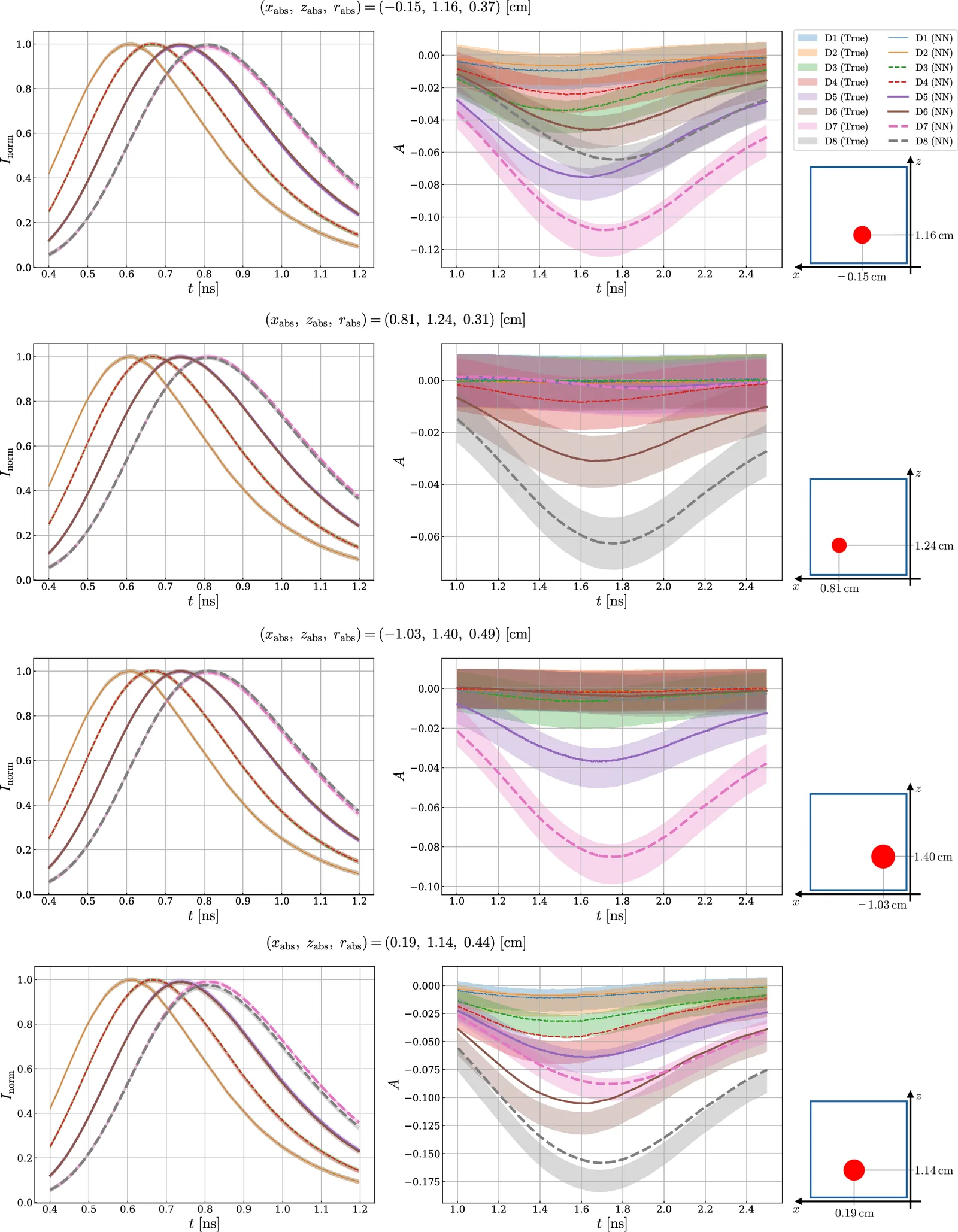
Four examples of signals predicted by the network trained with noiseless data. Time dependence of the normalized intensity $I_\textrm{norm}$ and the absorption measure *A* is shown in left and right panels, respectively. The used features $(x_\textrm{abs}, z_\textrm{abs}, r_\textrm{abs})$ are written above each plot. The solid lines are the signals predicted by the network, while the shaded regions are the true signal values *± 0.01*. Schematic illustrations of the absorbers (red circles) within the simulation box are shown next to each set of plots

Figure. 5 shows four examples of the time-resolved signals predicted using the network trained with noiseless data.

**Fig. 6:**
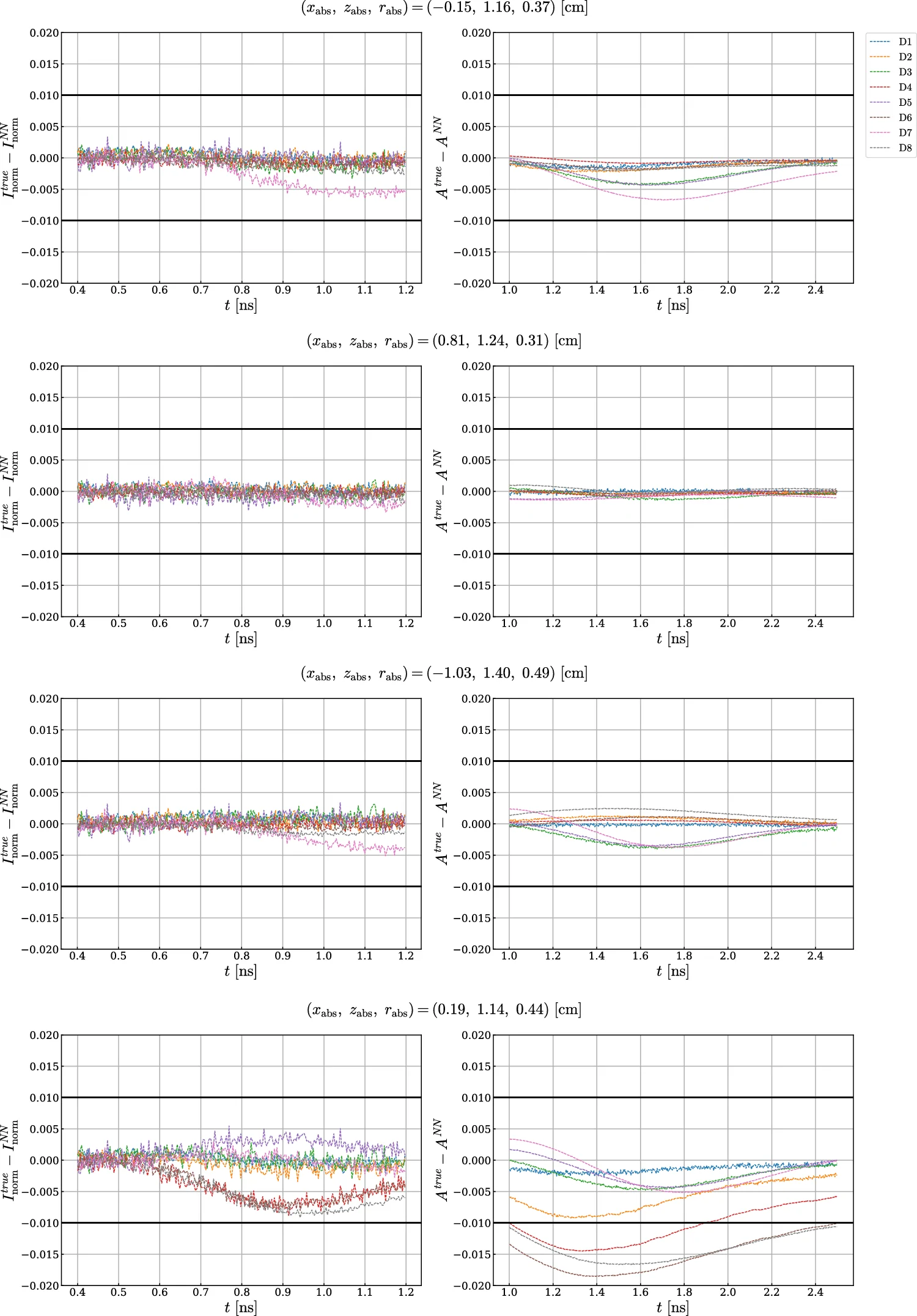
Four examples of the signal errors between the true values and those predicted by the network trained with noiseless data for $I_\textrm{norm}$ and *A*. The feature sets are identical to those shown in Fig. 5. The colored dashed lines indicate the time-dependent errors. The horizontal black solid lines at *± 0.01* denote the uncertainty range corresponding to the shaded regions in Fig. 5

To compare with the results obtained from the network trained with noise, we also plot the true signals with an uncertainty range of *± 0.01*, corresponding to the Gaussian noise added during training. We also present the time-dependent differences between the NN-predicted and true values of $I_\textrm{norm}$ and *A* in Fig. 6. For $I_\textrm{norm}^i(t)$, the true signal data generally exhibit a time shift proportional to the distances between the pulse injection point and the detectors. The values of the root mean squared error (RMSE, *σ *) computed with the predicted signal data and true data are *<0.01*, indicating that the network successfully captures the overall trends of the true signals. However, discrepancies close to 0.01 are observed around the peak values for some datasets. For example, for the case $(x_\textrm{abs}, z_\textrm{abs}, r_\textrm{abs}) = (0.19, 1.14, 0.44)\,\textrm{cm}$ (fourth row in Figs. 5 and 6), relatively larger deviations are observed at detectors D4, D6, and D8 for $t \gtrsim 0.8\,\textrm{ns}$. This suggests that while the overall RMSE is below 0.01, the network’s predictions can exhibit deviations of up to approximately 0.01 at specific times.

For $A^i(t)$, the network can approximate the time-resolved signals reasonably well for certain feature sets. In the case of $(x_\textrm{abs}, z_\textrm{abs}, r_\textrm{abs}) = (-0.15, 1.16, 0.37)\,\textrm{cm}$, the deviations between the predicted and true signals remain within 0.01. Similarly, for $(x_\textrm{abs}, z_\textrm{abs}, r_\textrm{abs}) = (0.81, 1.24, 0.31)\,\textrm{cm}$ and $(-1.03, 1.40, 0.49)\,\textrm{cm}$ (second and third rows from the top of Figs. 5 and 6), the discrepancies remain minimal over the entire time range. In contrast, for $(x_\textrm{abs}, z_\textrm{abs}, r_\textrm{abs}) = (0.19, 1.14, 0.44)\,\textrm{cm}$ (fourth row from the top of Figs. 5 and 6), the network fails to accurately reproduce the time-resolved signals. In this case, the predicted signals at detectors D4, D6, and D8 differ substantially from the true signals over most or all time points, with deviations exceeding 0.01. These results suggest that the network trained with noiseless data struggles to accurately reproduce the signals of $I_\textrm{norm}$ and *A* for some datasets. This behavior may be attributed to overfitting of the network to the training data. This behavior may be attributed to the high sensitivity of the time-resolved signals to absorbers located near the pulse injection point. To mitigate overfitting, increasing the number of training samples could be an effective strategy. However, the required dataset size for sufficient generalization remains unclear, and the total computational cost for generating such training data is also uncertain. Therefore, it may be more practical to adopt an alternative approach rather than relying solely on the network trained with noiseless data.

#### Neural network trained using data with noise

**Fig. 7:**
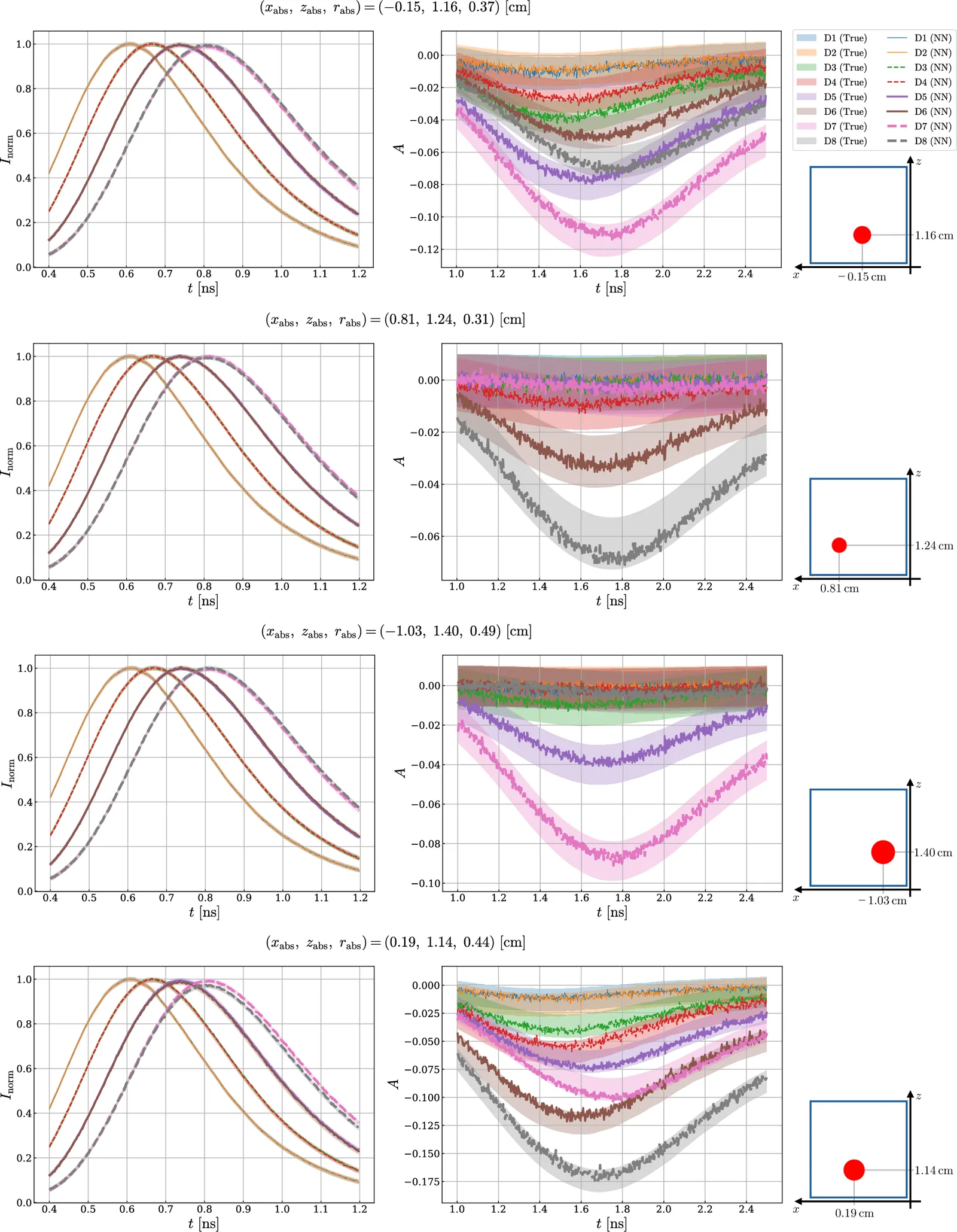
Same as Fig. 5 but the signals are predicted by the network trained using data with noise

**Fig. 8:**
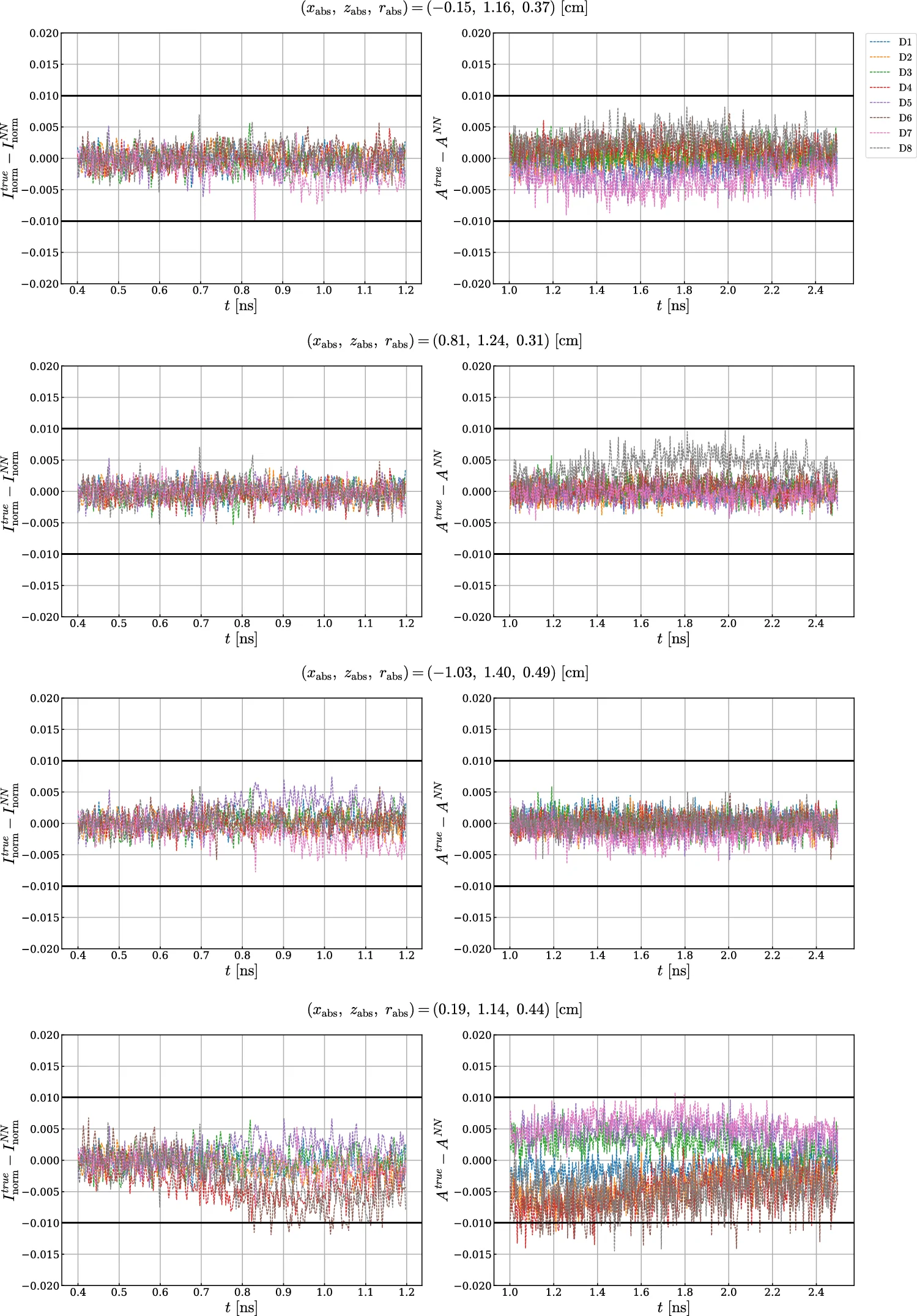
Same as Fig. 6 but the signals are predicted by the network trained using data with noise

In Fig. 7, we present the time-resolved signals predicted by the neural network trained on data obtained by increasing the training dataset tenfold through duplication and the addition of noise. The errors between the true and predicted values are also shown in Fig. 8. Compared to the predictions obtained from the network trained on noiseless data shown above, these results exhibit a significantly improved agreement with the true signals.

**Fig. 9:**
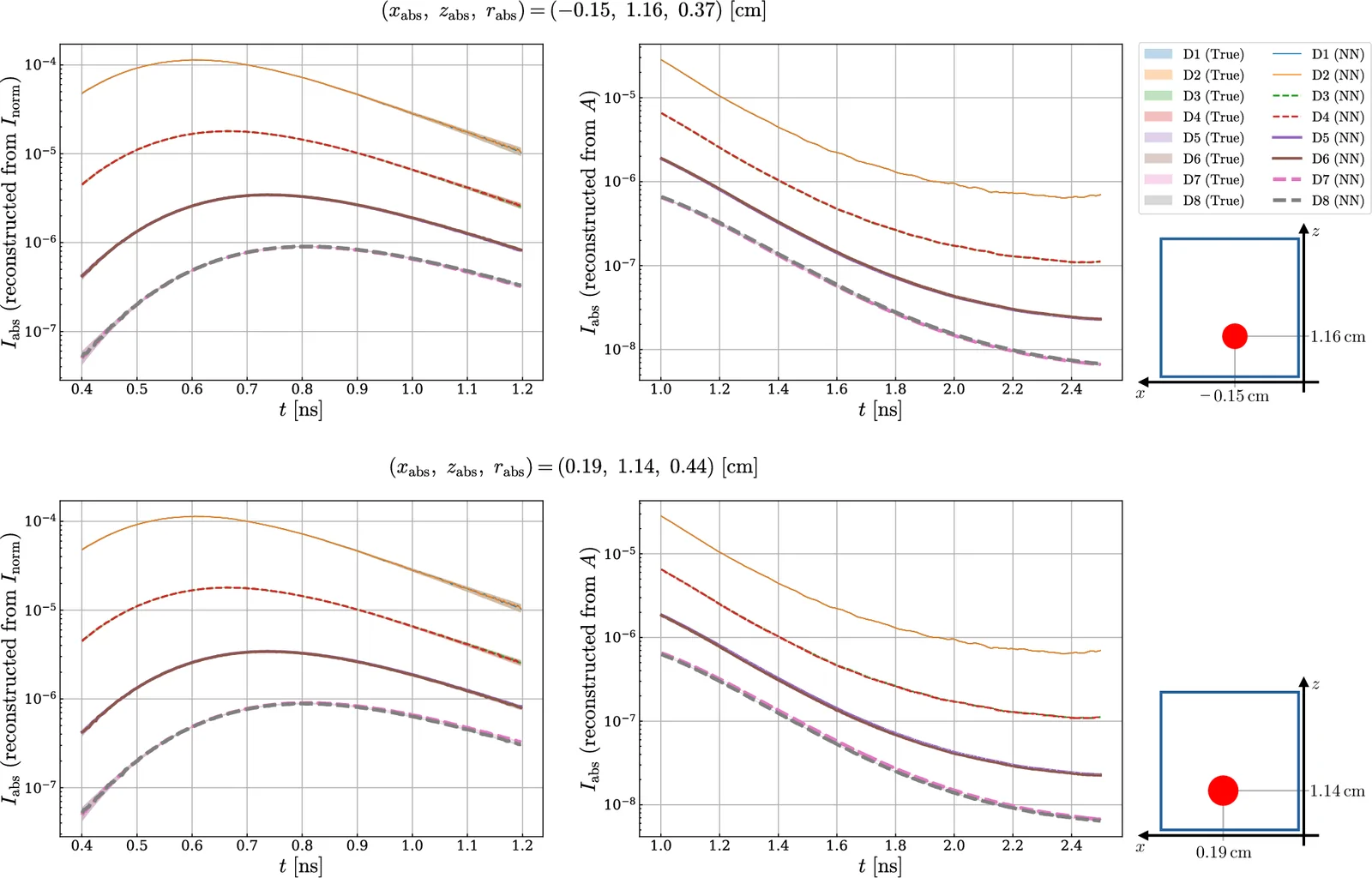
Time-resolved intensity $I_\textrm{abs}$ reconstructed from $I_\textrm{norm}$ (left) and *A* (right) using our NN together with Eqs. 3 and 4, respectively. The values of $I_\textrm{abs}$ are normalized to the maximum intensity of the input pulse. The detectors collect light incident within an opening angle of $\sim 15^\circ$ measured from the surface normal as in [20, 29]

For $I_\textrm{norm}$, the predicted signals closely match the true data, with deviations remaining within 0.01 for almost all time points In these predicted signals, fluctuations are observed, which may be due to the addition of noise in the training data. The magnitude of the fluctuations is typically within that of the added noise. For reference, we also present the time-resolved intensity at each detector, $I_\textrm{abs}^i(t)$, reconstructed using our NN. Since the relative variations in $I_\textrm{abs}^i(t)$ across different feature sets are less pronounced than those observed in $I_\textrm{norm}$ and *A*, we show only two representative cases: $(x_\textrm{abs}, z_\textrm{abs}, r_\textrm{abs}) = (-\,0.15, 1.16, 0.37)\,\textrm{cm}$ and $(0.19, 1.14, 0.44)\,\textrm{cm}$ (top and bottom panels of Fig. 7). The $I_\textrm{abs}^i(t)$ values reconstructed from *A* span a wider dynamic range than those reconstructed from $I_\textrm{norm}$.

The absorption measure *A* is predicted relatively accurately compared to the noiseless data case. In most cases, the predicted time-resolved signals of *A* are within the range of the added noise. Notably, when using the network trained with noise-augmented data, we obtain signals of *A* with smaller errors even for challenging feature sets. For example, for the feature set $(x_\textrm{abs}, z_\textrm{abs}, r_\textrm{abs}) = (0.19, 1.14, 0.44)\,\textrm{cm}$, which produced the poorest predictions with the noiseless network, the deviations of the signals are generally within 0.01. Therefore, the network trained with noise-augmented data exhibits markedly better performance than the one trained without noise. This improvement is likely attributable to the increased diversity and volume of training data achieved through duplication and noise injection.

**Fig. 10:**
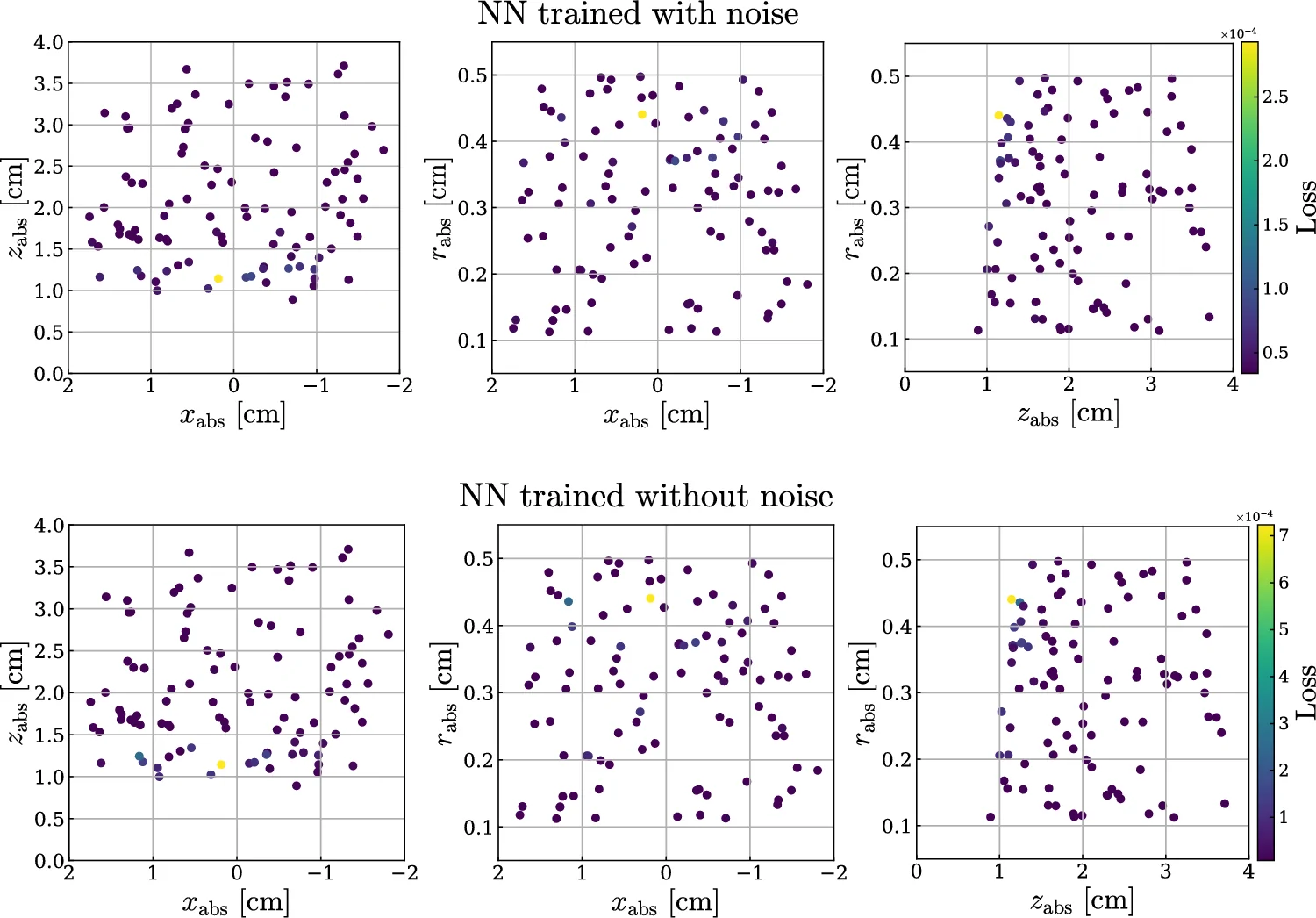
Loss distribution for test data in the feature space for both the networks trained using data with and without noise (top and bottom rows respectively). The panels show the $x_\textrm{abs}-z_\textrm{abs}$ planes (left), $x_\textrm{abs}-r_\textrm{abs}$ planes (center), and $z_\textrm{abs}-r_\textrm{abs}$ planes (right). Each marker means position in the feature space and is colored following their value of loss, which is computed from Eq. 5

To investigate the relationship between the feature space and the performance of the neural networks, we present the loss distribution for the test data in the feature space for both networks trained with and without noise in Fig. 10. The loss for each feature set in the test data is computed using Eq. 5.

One of the most notable findings is that the range of loss obtained from the network trained using data with noise is significantly smaller than that obtained from the network trained with noiseless data. The maximum loss from the former network is $2.9 \times 10^{-4}$, which is approximately 2.5 times smaller than that of the latter. The feature set producing the maximum loss in each network is $(x_\textrm{abs}, z_\textrm{abs}, r_\textrm{abs}) = (0.19, 1.14, 0.44)\,\textrm{cm}$, and its corresponding time-resolved signals are shown in the fourth row of both Fig. 5 and Fig. 7. This result suggests that the performance of a neural network model can be enhanced by replicating data and adding noise without the need to generate additional simulation data. However, the largest loss in each network is originated from the same feature set, indicating that factors other than the handling of training datasets may contribute to performance degradation. The feature of the highest loss is located near the pulse injection point at $(x, y) = (0.0, 0.0)\,\textrm{cm}$ on the *x*-*y* plane (Fig. 1). In the region near the point, small differences in the feature sets within this area can cause significant changes in the signal waveforms of $I_\textrm{norm}$ and *A*, particularly due to the increased absorption of light immediately after the pulse is injected. In other words, the time-resolved signals are highly sensitive to feature sets around the pulse injection point. Although there remain challenges in emulating time-resolved signals accurately, the network trained with data containing noise is able to produce time-resolved signals with lower loss, and the areas around higher loss are confined to regions near the pulse injection point. Given that these areas with higher losses in the feature space can be distinguished from those with lower loss, our neural network model could be effectively applied for the prompt diagnosis of anomalies like brain hemorrhage and tumor near the brain surface.

## Discussion

### Improving performance of the network

We have found that the neural network trained using data with noise can more accurately predict the time-resolved signals of the normalized intensity, $I_\textrm{norm}$, and the absorption measure, *A*, compared to the network trained with noiseless data. This result suggests that appropriate processing of the training data plays a crucial role in developing a better-performing network. Here, we discuss potential ways to further improve the network.

One of the simplest approaches is to run additional radiative transfer simulations with more feature sets, leveraging all available computational resources. In training a neural network for regression tasks, the network is designed to reproduce the training data, and having a more diverse set of feature sets enables the network to better predict time-resolved signals from unknown feature sets by interpolating between nearby values in the feature space.

However, indiscriminately running more simulations may not always be the most efficient use of limited computational resources. As shown in Fig. 10, the feature set that produce the largest loss is located at $(x_\textrm{abs}, z_\textrm{abs}, r_\textrm{abs}) \sim (0.19, 1.14, 0.44)\,\textrm{cm}$, which is close to the pulse injection point. Therefore, instead of generating simulation data simply, focusing on intensively sampling feature sets around the feature that produce the largest loss in the feature space and performing simulations with these sets could lead to an improved network with relatively fewer computational resources.

Although physics-informed or physics-constrained learning frameworks may further improve interpretability and generalization, explicitly embedding the physical structure of the RTE into the learning process is computationally impractical for the present problem. In addition, such an approach would undermine the primary objective of this work that develops a fast and accurate surrogate model for diagnostic applications based on detector-measured signals. We therefore adopt a data-driven neural network trained on physically accurate simulation data, while leaving physics-informed extensions to future work.

Another potential approach to improve the signal emulator is to develop an alternative neural network architecture. The hyperparameters for a neural network (e.g., learning rate, number of neurons, etc.) are also crucial factors that determine the performance of the network. However, there is a considerable degree of freedom when selecting the architecture and hyperparameters, making it challenging to identify the optimal model that minimizes the prediction losses. While improving the architecture or tuning hyperparameters could lead to better performance, such efforts often require extensive experimentation and fine-tuning. To address this limitation, Bayesian optimization provides an efficient framework for automatically identifying optimal hyperparameters, substantially reducing the required numerical experimentation [38, 39]. Therefore, combining additional training data from further radiation transfer simulations with Bayesian optimization for hyperparameter tuning offers a promising strategy to enhance the performance of the network.

### Application

We have developed a neural network model that produces the time-resolved signals of $I_\textrm{norm}$ and *A* from a given feature set of an absorber, aiming to quickly generate signals for diagnosing anomalies like brain hemorrhage and tumor near the brain surface. In terms of computational cost, our machine-learning model requires approximately $2\times 10^{-3}\,\textrm{sec}$ for a single inference on a CPU, whereas a full radiative transfer simulation typically takes several hours to complete. Therefore, by applying the neural network model, we can obtain a large amount of signal data from arbitrary feature sets in a much shorter time, although some errors are present.

For comparison with our neural network model, we have also employed Gaussian processes (GPs) as an alternative surrogate modeling approach. GPs provide both predictive mean functions and associated uncertainties based on the training data. We have found that although GPs yield well-fitted signals for certain feature sets within the test data, their prediction errors are generally much larger than those produced by our noise-augmented neural network model. For example, for the case of $(x_\textrm{abs}, z_\textrm{abs}, r_\textrm{abs}) = (0.19, 1.14, 0.44)\,\textrm{cm}$, the GP prediction error reaches 0.61, which is approximately 2000 times larger than the corresponding error from our network model. Moreover, each GP inference requires approximately $3.9\,\textrm{sec}$ on a CPU. Therefore, in terms of both predictive accuracy and computational efficiency, our neural network model outperforms the GP approach.

**Fig. 11:**
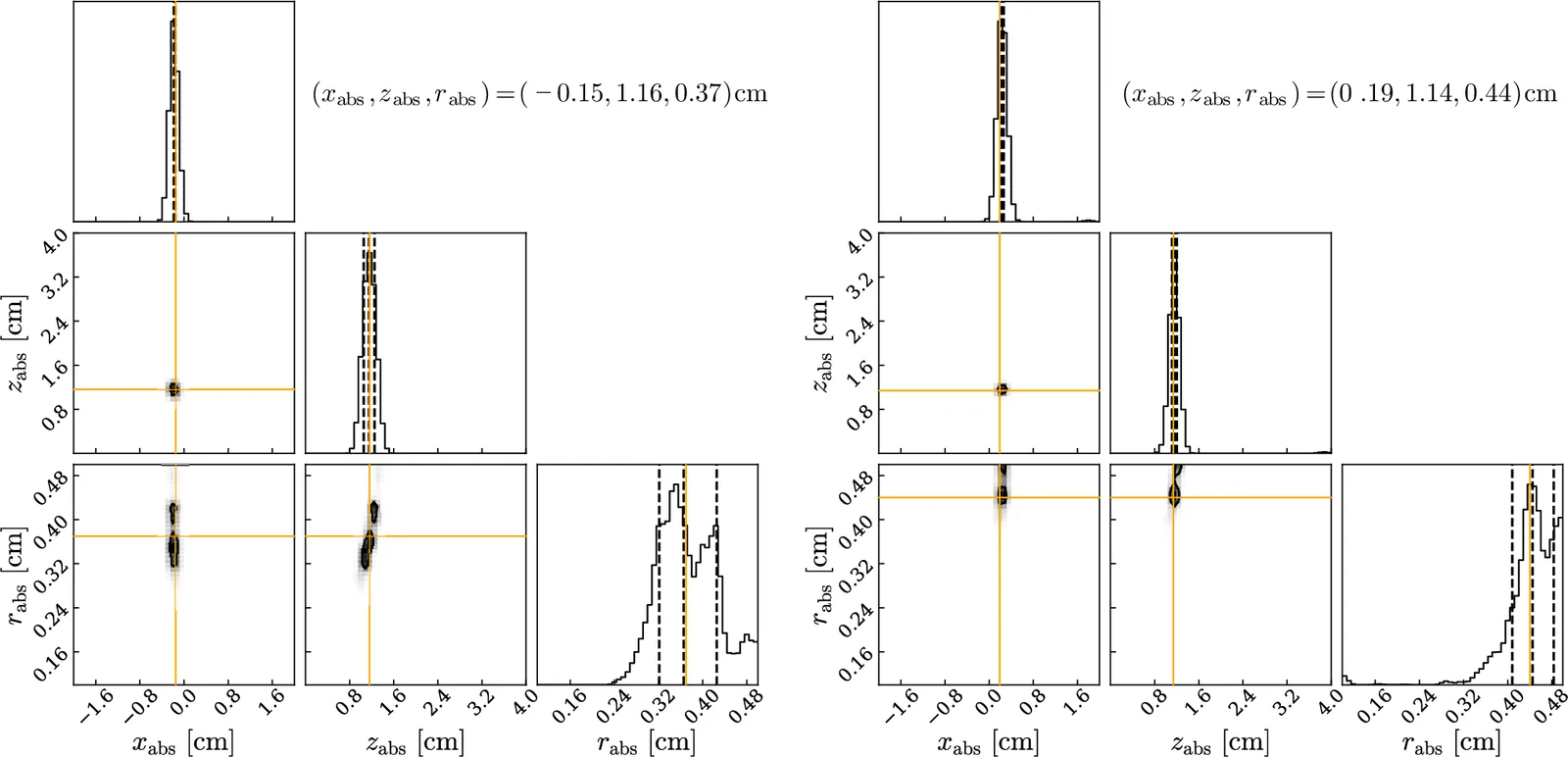
Posterior distributions of the absorber parameters $(x_\textrm{abs}, z_\textrm{abs}, r_\textrm{abs})$ inferred by inverse problem analysis using MCMC combined with our NN trained on noise-augmented data. Two representative cases are shown: $(x_\textrm{abs}, z_\textrm{abs}, r_\textrm{abs}) = (-0.15, 1.16, 0.37)\,\textrm{cm}$ and $(0.19, 1.14, 0.44)\,\textrm{cm}$ (corresponding to the top and bottom panels of Fig. 7). In the two-dimensional projections, the black solid contours denote the $68\,\%$ credible regions. In the one-dimensional marginalized distributions, the black dashed lines indicate the 16th, 50th, and 84th percentiles. The orange solid lines mark the true parameter values, which are shown at the top right of each corner plot

One potential application of this network is diagnosing with MCMC methods, which allow for the estimation of a probability density function through random sampling. By comparing observed time-resolved signals with those predicted by the network, an MCMC algorithm can determine the probability distribution of features that best reproduce the observed signals. We demonstrate inverse problem analysis by combining MCMC with our NN model trained on noise-augmented data to infer absorber parameters from observed time-resolved signals, using the *emcee* package [40]. Figure 11 presents the posterior distributions of $x_\textrm{abs}$, $z_\textrm{abs}$, and $r_\textrm{abs}$ obtained from this analysis for two representative cases, $(x_\textrm{abs}, z_\textrm{abs}, r_\textrm{abs}) = (-\,0.15, 1.16, 0.37)\,\textrm{cm}$ and $(0.19, 1.14, 0.44)\,\textrm{cm}$ (top and bottom panels of Fig. 7), shown as corner plots. For the case $(-\,0.15, 1.16, 0.37)\,\textrm{cm}$, the absorber position is accurately recovered, and the radius is also reasonably well constrained, although its posterior distribution is broader than those of the position parameters. The true values lie within the $68\,\%$ credible regions of the two-dimensional posteriors and between the 16th and 84th percentiles of the one-dimensional marginals. The corresponding uncertainties in the inferred radius are approximately $0.05\,\textrm{cm}$ and $0.06\,\textrm{cm}$. For the second case, $(0.19, 1.14, 0.44)\,\textrm{cm}$, all three parameters are likewise accurately estimated. The true values fall within the $68\,\%$ credible regions of the joint distributions, and although the one-dimensional posterior for the radius is relatively broad, its maximum closely coincides with the true value. The uncertainties in the inferred radius are approximately $0.03\,\textrm{cm}$ and $0.04\,\textrm{cm}$. These results demonstrate that, even when the predicted signals exhibit errors comparable to $\pm \,0.01$ (Figs. 7 and 8), the absorber parameters can still be robustly inferred by combining MCMC with our NN. This highlights the potential of our approach as an efficient surrogate-based framework for inverse problem analysis. However, the current feature space is limited to the horizontal direction $x_\textrm{abs}$, depth $z_\textrm{abs}$, and radius $r_\textrm{abs}$, with the fixed vertical direction $y_\textrm{abs}$, absorption coefficient $\mu _\textrm{a}$, and the assumption of a single spherical absorber in the simulation box. To enable more detailed anomaly diagnoses, additional factors such as $y_\textrm{abs}$, $\mu _\textrm{a}$, the shape of the absorber, and the presence of multiple absorbers in the box should be considered. In future work, we plan to develop an enhanced neural network model that incorporates these factors, as well as an algorithm combining this model with MCMC for improved diagnosis in brain anomalies.

Our current neural network model is specifically designed for brain tissue optical properties and is therefore limited to applications involving brain structures. In principle, however, individual models could be developed for other tissue types, including those with multiple or more complex structural compositions in different regions of the body.

## Conclusion

We have developed a simple neural network model that reproduces time-resolved signals of the normalized intensity, $I_\textrm{norm}$, and the absorption measure, *A*, at eight detectors, based on three features of the absorber: horizontal direction $x_\textrm{abs}$, depth $z_\textrm{abs}$, and radius $r_\textrm{abs}$, which simulate anomalies such as brain hemorrhage and tumor near the brain surface. To obtain the training data, we conduct radiative transfer simulations using the TRINITY code [20], which provides highly accurate time-resolved signal data by directly solving the time-dependent RTE. For the simulations, we generate 640 feature sets using LHS, which enables sampling of parameters with minimal bias. Our network employs deep multi-task learning and consists of one input layer, one shared hidden layer, and task-specific layers, each containing three hidden layers and one output layer for $I_\textrm{norm}$ and *A* at each detector.

We have found that the network, trained using data generated by duplicating and adding noise, can predict time-resolved signals with errors that are at most comparable to the size of the added noise. This suggests that our network can produce time-resolved signals both precisely and quickly. In contrast, when the network is trained without these data treatments, it cannot achieve such accuracy. Thus, pre-processing of the training data is crucial for improving network performance. However, when the absorber is located near the pulse injection point, the network performance deteriorates, with relatively poor regression results. This may be due to the fact that signals are particularly sensitive to the absorption of light immediately after the pulse is injected. Therefore, the network performance could be further enhanced by conducting additional simulations for feature sets near the pulse injection point.

Since the network can quickly generate time-resolved signals, it holds promise for use in anomaly diagnosis, especially when combined with MCMC methods. Since the feature space in this study is limited, there is a need to develop a network model trained with a broader feature space for clinical applications. Nevertheless, our research demonstrates the potential for a new diagnostic approach for anomalies using neural networks.
